# Case Report: Pulmonary color Doppler ultrasonography features of congenital tuberculosis in a preterm infant

**DOI:** 10.3389/fped.2025.1639412

**Published:** 2025-10-23

**Authors:** Di Zhou, Wanxu Guo, Di Ma, Yunfeng Zhang

**Affiliations:** Department of Neonatology, The Second Hospital of Jilin University, Changchun, Jilin, China

**Keywords:** *Mycobacterium tuberculosis*, congenital tuberculosis, color Doppler ultrasound, neonatal respiratory distress syndrome (NRDS), preterm infant

## Abstract

This study explores the color Doppler ultrasonography features of congenital tuberculosis in preterm infants through a detailed analysis of a case from February 2025, including clinical, NGS, and sonographic data. The findings identify specific vascular signatures that facilitate early diagnosis, offering a valuable reference for clinicians encountering similar cases. High-frequency probe ultrasonography was used to characterize pulmonary lesions and differentiate tuberculosis from bronchopulmonary dysplasia (BPD) and neonatal respiratory distress syndrome (NRDS). A male infant born at gestational age of 28 weeks (birth weight: 1,180 g) presented with respiratory distress and cyanosis (Apgar score: 7 at 1 min). Initial ultrasonography revealed multiple confluent B-lines in both lungs. At 60 days postpartum, ultrasonography showed thickened pleura, scattered subpleural consolidations, atelectasis in the lower lobes, and hyperechoic fragments moving with respiration. First-line anti-TB regimen, isoniazid, rifampin, and pyrazinamide were administered and serial monitoring via color Doppler ultrasound and chest CT demonstrated favorable disease control. Main take-away lessons: Pulmonary color Doppler ultrasonography, combined with clinical history, can aid in the diagnosis of congenital tuberculosis, and congenital TB should be considered in preterm infants with refractory pneumonia. Additionally, NGS is a valuable tool for rapid pathogen identification.

## 1 Introduction

Congenital tuberculosis (TB) is a rare yet severe form of *Mycobacterium tuberculosis (M. tuberculosis)* infection. Fetuses may acquire the disease via transplacental hematogenous spread or through aspiration/ingestion of contaminated amniotic fluid during delivery (1).

According to 2022 global statistics, there were 10.6 million new TB cases worldwide (2). China reported 748,000 cases (7.1% of the global total), ranking third among the 30 high-TB-burden countries, with adult females accounting for 32% of cases (3). The WHO's 2021 Global TB Report estimated 9.9 million incident TB cases globally in 2020, including 1.09 million pediatric cases and approximately 200,000 child deaths (4). Currently, there is no systematic registry or large-scale cohort study specifically targeting congenital tuberculosis both domestically and internationally (5, 6). Relevant data are extremely scarce, mainly relying on case reports and regional retrospective studies. According to the WHO's Global Tuberculosis Report 2024, the estimated number of global tuberculosis cases was 10.8 million in 2023 (7). It is estimated that there were about 1.3 million cases among children and adolescents (8). Due to the challenges in accurate diagnosis, there is a significant gap in case detection and reporting, especially among children under 5 years old (9). In addition, about 50% of suspected cases were not reported in this age group (9). The global number of congenital tuberculosis cases is less than 12,500, equivalent to 0.3 per 100,000 live births (10).

Current TB diagnostic methods include acid-fast staining, molecular assays, and culture (11). Next-generation sequencing (NGS) (12, 13), an emerging molecular technique, demonstrates significant advantages in pathogen detection and antimicrobial resistance profiling—particularly for mycobacterial diseases (14). Additionally, color Doppler ultrasound applied in pulmonary TB reduces radiation exposure and alleviates patients' financial burdens (15).

This article presents a congenital TB case diagnosed through lung color Doppler ultrasound combined with NGS. We analyze sonographic features to improve diagnostic accuracy in preterm infants and discuss relevant literature.

## 2 Case description

### 2.1 Infant clinical data

A gestational age of 28-week male (1,180 g) was delivered via cesarean section due to maternal fever and preterm premature rupture of membranes (PPROM). At birth, he exhibited respiratory distress (Apgar: 7 at 1 min, 9 at 5 min). Initial chest x-ray showed reduced lung transparency; arterial blood gas analysis results revealed metabolic acidosis (pH 7.077, pCO₂ 67.5 mmHg, pO_2_ 81.6 mmHg, Na^+^ concentration 134.0 mmol/L, K^+^ concentration 3.3 mmol/L, Ca^2+^ concentration 1.3 mmol/L, serum glucose levels 2.9 mmol/L, lactic acid 1.7 mmol/L, HCO_3_^−^ 19.4 mmol/L, base excess −12.0 mmol/L, hemoglobin 172.0 g/L). He was diagnosed with NRDS, electrolyte imbalances, and metabolic acidosis. The infant's mother had a normal physical recovery after childbirth and underwent a pulmonary CT scan, which showed no abnormalities. She was breastfeeding the infant.

### 2.2 Maternal clinical data

The mother had a history of infertility (suspected tubal TB) and fever (38°C) before delivery. The infant presented with elevated infection markers after birth. Antibacterial treatment proved ineffective and resulted in a prolonged clinical course. After 60 days of hospitalization, a detailed maternal medical history revealed that the mother had visited a gynecologist prior to pregnancy. Relevant imaging studies showed wire-like changes and rigidity in the fallopian tube lumen, which were considered indicative of tuberculosis-related damage. Since the disease was in an inactive phase at the time, a tuberculin test was not recommended. The mother was strongly suspected of having had pelvic tuberculosis in the past. Pathological examination of the placenta showed no abnormalities.

## 3 Diagnostic assessment & therapeutic intervention

### 3.1 Case presentation

Initial treatment included surfactant therapy, broad-spectrum antibiotics, and mechanical ventilation. However, the patient's condition failed to improve significantly. On day 20 of hospitalization, the patient developed a persistent fever. A chest x-ray revealed patchy infiltrates, raising the suspicion of hospital-acquired pneumonia. Despite escalating the antibiotic regimen, the clinical symptoms and radiographic findings showed no resolution.

### 3.2 Definitive diagnosis

**Figure 1:**
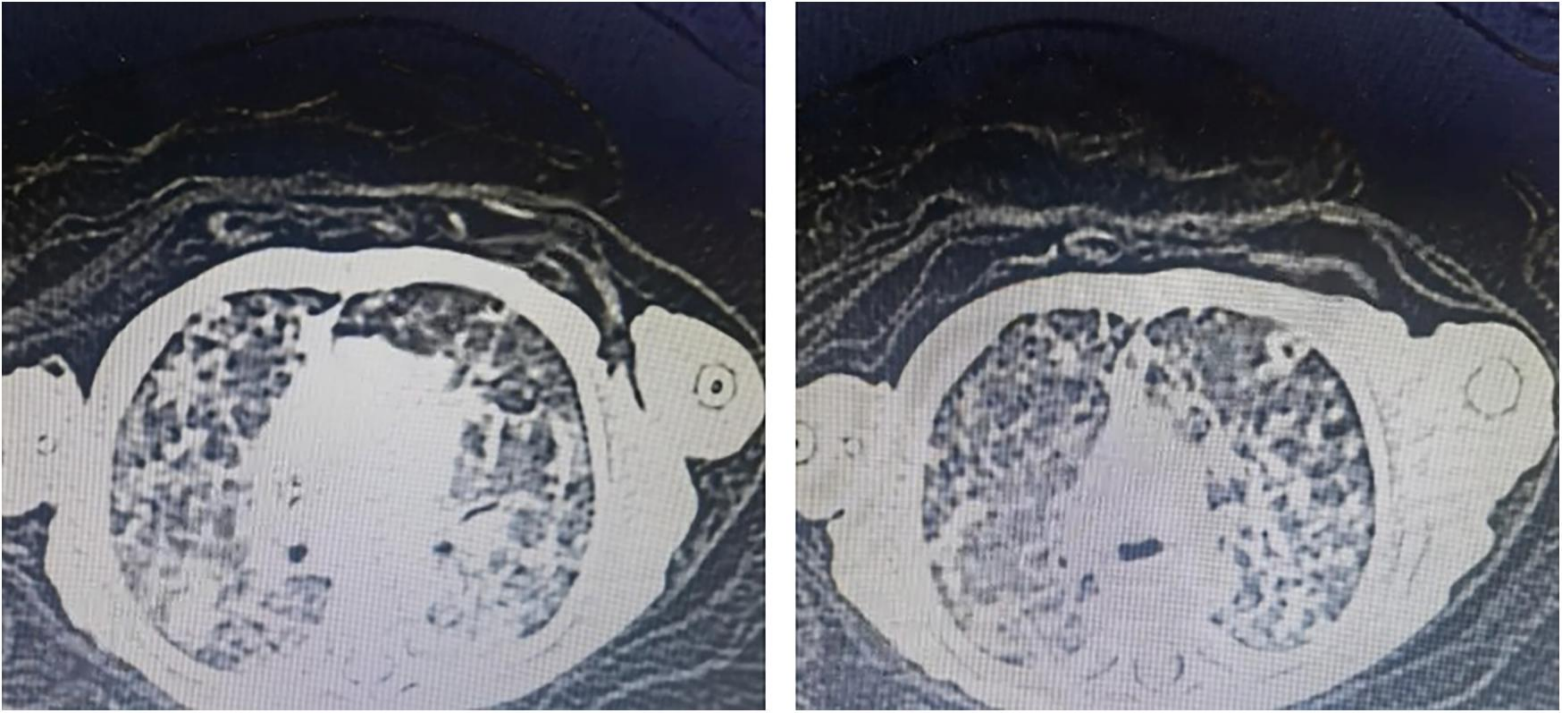
CT image showing miliary TB.

The infant had high levels of infection markers after birth, and the antibacterial treatment was not very effective, with a longer course of treatment. Blood specimen was used to perform NGS detection of *M. tuberculosis* complex at day 60. After the diagnosis of tuberculosis was confirmed, the antitubercular treatment was effective. CT revealed miliary TB (Figure 1).

### 3.3 Ultrasonography findings

**Figure 2:**
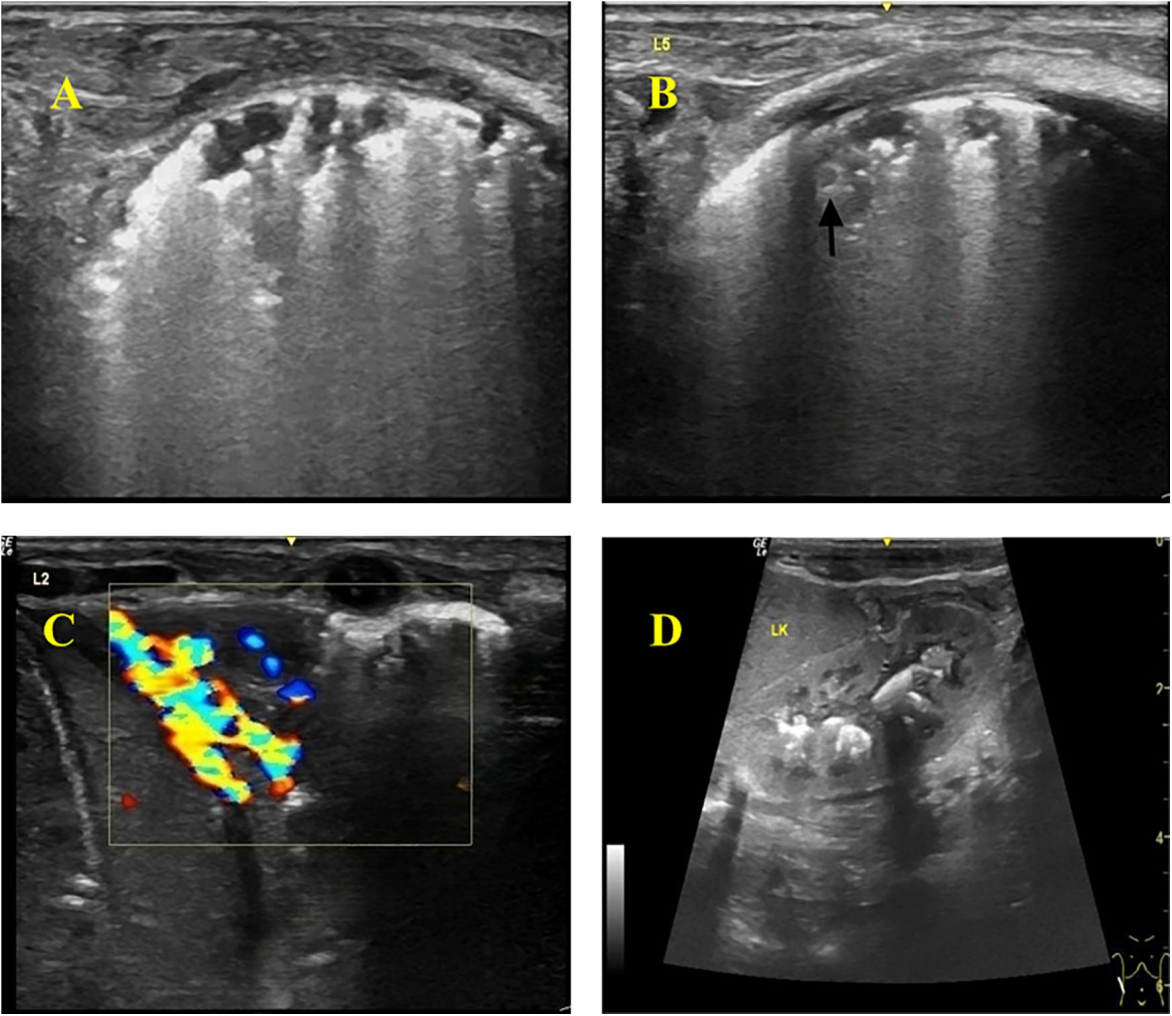
Color Doppler ultrasonography of the infant. **(A)** Thickened and hyperechoic pleural lines in both lungs, with partial discontinuity. **(B)** Multiple discontinuous small subpleural hypoechoic lesions (*ragged cloth sign*) with scattered hyperechoic patchy areas (*fragmentation sign*) at the margins. Nodular changes within some consolidated areas. **(C)** Wedge-shaped consolidation in the lower lobes of both lungs, with heterogeneous echogenicity and small hypoechoic areas, showing rich blood flow signals on Doppler. **(D)** Abdomen (Color Doppler Ultrasound): Hepatomegaly and splenomegaly. Calcifications visible in the renal pelvises and calyces of both kidneys.

Thickened, irregular pleura with “shred sign” (hyperechoic fragments). Subpleural consolidations with heterogeneous echotexture and vascularity. Multiple solid lesions of varying sizes were visible in the subpleural areas of both lungs, with the larger ones mainly located in the lung apices and bases, involving 2–3 intercostal spaces. The pleura was thickened at the sites of consolidation, with a thickness of about 2 mm. Blood flow signals were detected in the consolidated areas, with a resistance index (RI) of 0.86–0.88. Hepatosplenomegaly and renal calcifications were also identified on abdominal ultrasound (Figure 2).

The ultrasound settings used in this study. Machine: GE LOGIQ e; Transducer: L4-12t linear probe; Frequency: 12 MHz; Depth: 3–4 cm; Overall Gain: 72%; TGC: Set in a left-low–right-high pattern (approx. 45 dB on the right); Tissue Harmonic Imaging: Off; Compound Imaging: Off; Focal Zone: One zone positioned at the pleural line.

### 3.4 Therapeutic interventions

The management of congenital tuberculosis includes anti-tuberculosis pharmacotherapy, isolation measures, symptomatic/supportive care, and close monitoring of disease progression. Currently, pharmacotherapy remains the primary treatment for tuberculosis. The standard first-line regimen consists of isoniazid + rifampin + pyrazinamide, with a prolonged treatment course typically lasting 6–9 months for newly diagnosed patients (16, 17).

In this case, the infant was diagnosed with miliary tuberculosis and would ordinarily require transfer to a specialized TB hospital. However, as a 28-week preterm neonate with low body weight (at 60 days postnatal age) and ongoing ventilator dependence, transfer was considered unfeasible. Consequently, the patient received isolated treatment in our unit with the following regimen: Isoniazid: 17.9 mg once daily; Rifampin: 17.9 mg once daily; Pyrazinamide: 35.8 mg once daily; Serial monitoring via color Doppler ultrasound and chest CT demonstrated favorable disease control. It should be noted that although pelvic tuberculosis is non-infectious and newborns generally do not transmit tuberculosis, isolation measures were still implemented as a precaution based on the maternal pregnancy history and the infant's treatment response.

A timeline with relevant data from the episode of care is shown in Table 1.

**Table 1:** Timeline showcasing relevant data from the episode of care.

Timeline	Clinical event	Treatment measures
At birth	28-week preterm, low birth weight, pulmonary dysplasia	Endotracheal surfactant administration 240mg
Day 20 of admission	Fever, patchy infiltrates on CXR	Diagnosed as neonatal pneumonia
Days 20–60	Recurrent fever, elevated inflammatory markers	2× TB skin tests: negative Advanced antibiotics + IVIG, poor response
Day 60	Treatment failure	NGS: *Mycobacterium tuberculosis* complex CT: Miliary TB pattern
Post-diagnosis	Ultrasound findings: Rag sign, Debris sign Hepatosplenomegaly, renal calcifications	First-line anti-TB regimen Isoniazid: 17.9 mg once daily; Rifampin:17.9 mg once daily; Pyrazinamide: 35.8 mg once

## 4 Discussion

*Mycobacterium tuberculosis* is an aerobic bacterium, while the intrauterine environment is relatively hypoxic, which is unfavorable for its growth. After birth, *Mycobacterium tuberculosis* requires a certain incubation period before symptoms appear, typically manifesting within 2–3 weeks after delivery. Early symptoms include fever, tachypnea, apnea, weight loss, irritability, abdominal distension, feeding difficulties, and hepatosplenomegaly (18). Neonatal tuberculosis progresses rapidly, with a mortality rate as high as 40%–60%. Without timely diagnosis and treatment, the mortality rate increases further (19). Early diagnosis can significantly improve prognosis.

In this case, the infant's blood tests indicated elevated infection markers and recurrent fever. Despite treatment with high-dose antibiotics and intravenous immunoglobulin therapy, no significant improvement was observed. The infant underwent extensive diagnostic workup without a definitive diagnosis. Chest CT and *Mycobacterium tuberculosis* infection tests during hospitalization showed no obvious abnormalities, making it difficult to distinguish from traditional Neonatal Respiratory Distress Syndrome (NRDS). However, next-generation sequencing (NGS) and color Doppler ultrasound ultimately confirmed the diagnosis of miliary tuberculosis.

### 4.1 Characteristic color Doppler ultrasound findings in congenital tuberculosis

When *Mycobacterium tuberculosis* invades lung tissue, it triggers pathological reactions such as exudation, proliferation, and necrosis. The alveolar air spaces are replaced by pathological tissue, and when the pleura is involved, color Doppler ultrasound may reveal lung consolidation, subpleural nodules, pleural thickening, and pleural effusion (20).

In this case, the infant presented with recurrent fever and poor response to antibiotics. Chest x-ray showed prominent lung markings, prompting a lung color Doppler ultrasound, which revealed: (1) Abnormal pleural line: appeared rough, irregular, and hyperechoic, with posterior confluent or dense B-lines, some discontinuous with thin pleural segments. (2) Multiple irregular consolidations: subpleural hypoechoic consolidations of varying sizes and shapes were observed. Smaller lesions were highly irregular with fragmented hyperechoic borders, while larger wedge-shaped consolidations were seen in the apical and lower lobes, also with fragmented hyperechoic edges. The consolidations showed minimal aeration, heterogeneous echogenicity (patchy hypoechoic areas), and grade 2 blood flow signal. (3) Nodular changes: Nodular lesions with slightly hyperechoic rims were detected within subpleural consolidations.

Abdominal color Doppler ultrasound revealed hepatosplenomegaly and multiple hyperechoic foci in the renal pelvis, but no enlarged mediastinal lymph nodes.

While our study highlights the pivotal role of ultrasound in suggesting the diagnosis of congenital TB, it is crucial to acknowledge that a definitive diagnosis relies on a comprehensive approach rather than on microbiological tests alone. The integration of clinical symptomatology, detailed maternal history (including the type and activity of maternal TB disease), histopathological examination of the placenta, and developmental data of the infant is imperative, especially in cases where AFB smear, culture, or molecular tests are negative or unavailable. Our ultrasound findings should therefore be interpreted as a key component within this broader diagnostic framework, prompting clinicians to pursue a full array of investigations to confirm or exclude the disease.

### 4.2 Differential diagnosis of congenital tuberculosis vs. other neonatal lung diseases

Neonatal tuberculosis lacks specific clinical manifestations, particularly in preterm infants, where it can mimic NRDS or Bronchopulmonary Dysplasia (BPD), leading to delayed diagnosis and treatment. While chest x-ray has low specificity and sensitivity in early tuberculosis, chest CT provides more detailed imaging but has limitations in preterm infants.

Bedside lung ultrasound has gained widespread use in neonatal lung disease diagnosis and can partially replace traditional x-rays. This infant, born at 28 weeks, received surfactant therapy and antibiotics but remained febrile. Lung ultrasound was performed to differentiate between NRDS and BPD (Table 2).

**Table 2:** Differential diagnosis of lung diseases by ultrasound.

Ultrasonic features	Congenital tuberculosis	NRDS	BPD
Pleural line	Rough, irregular, hyperechoic, with segmental thinning or interruption	Blurry, absent, with visible “double lung point”	Early rough/blurry; late moth-eaten or cystic
B-lines	Fused or dense B-lines, unevenly distributed	Diffuse fused B-lines (pulmonary edema)	Early predominantly fused B-lines; late moth-eaten or cystic changes
Consolidation area	Multiple subpleural, extremely irregular in shape, with “shredded” edges; heterogeneous internal echoes, with more common hypoechoic areas	Homogeneous wedge-shaped consolidation with clear boundaries; obvious “snowflake” or “speckled” air bronchograms within	Early rare consolidation; late fibrosis with cord-like hyperechoic areas
Air bronchogram	Sparse or absent, with fragmented hyperechoic edges	Dense, uniform, punctate-linear air bronchograms	Absent (early) or cord-like (fibrotic phase)
Doppler flow signals	Abundant blood flow within the consolidation (≥2 grades)	Absent or minimal blood flow	Reduced blood flow in late fibrotic areas
Others	Hepatosplenomegaly and renal calcifications suggesting dissemination	Being associated with pleural effusion	–

#### 4.2.1 Differentiation from neonatal respiratory distress syndrome (NRDS)

NRDS is characterized by lung consolidation with air bronchograms, pleural line abnormalities (loss of A-lines), double lung points, and pleural effusion (21). It results from surfactant deficiency, leading to hyaline membrane formation and atelectasis (22).

In contrast, congenital tuberculosis consolidations arise from tuberculous exudation, proliferation, and necrosis. Key ultrasound differences: (1) NRDS: Dense, uniform air bronchograms (snowflake-like, dotted, or linear) with clear boundaries (23). (2) Tuberculosis: Irregular consolidations with fragmented borders, heterogeneous echogenicity, and minimal air bronchograms.

This infant initially resembled NRDS, but lung ultrasound revealed atypical inflammatory signs. Despite broad-spectrum antibiotics, persistent fever prompted further investigation. Upon reviewing the mother's history, a suspected tubal tuberculosis infection was noted. NGS later confirmed *MS* infection.

#### 4.2.2 Differentiation from bronchopulmonary dysplasia (BPD)

Early BPD lacks specific ultrasound findings, but late-stage BPD shows fibrosis and cystic changes (24). This infant, hospitalized for 3 months, required BPD exclusion.

BPD ultrasound findings: (1) Non-specific pleural line abnormalities (roughness, blurring, discontinuity). (2) Specific “moth-eaten” or cystic pleural changes in advanced stages (24).

Tuberculosis ultrasound findings: (1) Rough, irregular, hyperechoic pleural line (non-specific). (2) Multiple subpleural irregular consolidations (distinct from BPD).

Early BPD may mimic tuberculosis with confluent B-lines (pulmonary edema), but late-stage BPD's “moth-eaten” pleura helps differentiation.

## 5 Conclusion

Congenital tuberculosis lacks specific signs, and delayed treatment leads to high mortality. Bedside ultrasound, being radiation-free and portable, plays a crucial role in neonatal diagnosis. This report details color Doppler ultrasound features in a miliary tuberculosis case, highlighting subtle differences that may aid early diagnosis and improve outcomes. This study is a case report, LUS successfully visualized the lesion adjacent to the costophrenic angle; however, regions masked by bone could not be assessed. and more related cases are needed for multicenter prospective studies.

## 6 Key takeaways

(1) Congenital TB should be considered in preterm infants with refractory pneumonia.
(2) Pulmonary ultrasonography can differentiate TB from NRDS/BPD.
(3) NGS is a valuable tool for rapid pathogen identification.

## Data Availability

The original contributions presented in the study are included in the article/Supplementary Material, further inquiries can be directed to the corresponding author.
